# From paradox to biology: shared genetic architecture underlies the inverse association between smoking and Sjögren’s disease

**DOI:** 10.1093/rheumatology/keag385

**Published:** 2026-07-24

**Authors:** Stefanie van der Merwe, Benjamin Zuckerman, Weijie Liu, Wenjie Cheng, Sizheng Steven Zhao

**Affiliations:** Centre for Musculoskeletal Research, Division of Musculoskeletal and Dermatological Science, School of Biological Sciences, Faculty of Biological Medicine and Health, The University of Manchester, Manchester Academic Health Science Centre, Manchester, UK; Centre for Musculoskeletal Research, Division of Musculoskeletal and Dermatological Science, School of Biological Sciences, Faculty of Biological Medicine and Health, The University of Manchester, Manchester Academic Health Science Centre, Manchester, UK; Department of Inflammation Biology, Centre for Rheumatic Diseases, King’s College London, London, UK; Centre for Musculoskeletal Research, Division of Musculoskeletal and Dermatological Science, School of Biological Sciences, Faculty of Biological Medicine and Health, The University of Manchester, Manchester Academic Health Science Centre, Manchester, UK; Centre for Musculoskeletal Research, Division of Musculoskeletal and Dermatological Science, School of Biological Sciences, Faculty of Biological Medicine and Health, The University of Manchester, Manchester Academic Health Science Centre, Manchester, UK; Dermatology Center, Xinhua Hospital, Shanghai Jiaotong University School of Medicine, Shanghai, China; Centre for Musculoskeletal Research, Division of Musculoskeletal and Dermatological Science, School of Biological Sciences, Faculty of Biological Medicine and Health, The University of Manchester, Manchester Academic Health Science Centre, Manchester, UK; NIHR Manchester Biomedical Research Centre, Manchester University NHS Foundation Trust, Manchester, UK

**Keywords:** Sjögren’s disease, smoking, Mendelian randomization, pleiotropy, colocalization, genetic epidemiology

## Abstract

**Objectives:**

Smoking has been consistently associated with a paradoxically lower risk of Sjögren’s disease (SjD). We used genetic approaches to investigate whether this association reflects a causal effect of smoking or shared genetic architecture, and to identify shared loci that may help explain this paradox and inform disease biology.

**Methods:**

We applied an integrated cross-trait genetic framework using genome-wide association data for smoking initiation and SjD. Conventional Mendelian randomization (MR) was used as an initial test of association, followed by genome-wide causal modelling (CAUSE and latent causal variable analysis) to distinguish directional causality from shared genetic architecture. Shared loci were identified using cross-trait pleiotropy analysis, refined through colocalization, and further explored using proteome-informed mediation analyses.

**Results:**

Conventional MR showed an inverse association between genetic liability to smoking initiation and SjD risk (OR 0.53, 95% CI 0.34–0.84). However, genome-wide modelling did not support a causal effect and instead suggested the association was driven by shared genetic influences. Cross-trait pleiotropy analysis identified 1536 variants across 11 loci, with colocalization supporting shared causal variants at five regions (3q13.32, 2q22.3, 8p21.2, 14q24.3 and 5q34). These loci implicated pathways related to neuroimmune signalling, epithelial organization and interferon regulation. Proteome-informed analyses identified no circulating proteins that robustly mediated the smoking–SjD relationship.

**Conclusions:**

The inverse smoking–SjD association likely reflects shared genetic liability rather than a protective causal effect. These findings highlight biological pathways relevant to SjD pathogenesis and emphasize the importance of cautious interpretation of MR estimates when traits share genetic architecture.

Rheumatology key messages

- Inverse smoking-Sjögren’s association reflects shared genetics, not causal protective effects.
- Genome-wide analyses show no directional causality between smoking initiation and Sjögren’s disease.
- Shared loci implicate neuroimmune, epithelial and interferon pathways in Sjögren’s pathogenesis.

## Introduction

Paradoxical associations in epidemiology are often dismissed as artefacts of bias, confounding or reverse causation. However, some paradoxes can lead to meaningful biological insight when dissected using appropriate methods. Examples include the opposing effects of TNF inhibition in RA and multiple sclerosis [1, 2], and the inverse association between smoking and ulcerative colitis [3], both of which informed disease mechanisms. The challenge is therefore not in identifying paradoxes, but in extracting biological insight from them.

Sjögren’s disease (SjD) presents one such opportunity. Observational studies have reported that smoking is associated with a reduced risk of SjD, despite smoking being a well-established risk factor in most autoimmune diseases [4]. This paradox has been consistently replicated across populations yet remains unexplained, raising the possibility that smoking-related biology, or pathways genetically linked to smoking behaviours, may illuminate disease-relevant mechanisms. This is particularly pertinent because SjD lacks effective disease-modifying therapies and management is largely symptomatic [5]. Although the SjD therapeutic landscape is evolving, with several targeted agents showing promise in recent or ongoing late-phase trials, effective disease-modifying options remain limited in routine clinical practice. This supports continued investigation of disease-relevant pathways beyond established immune targets.

Mendelian randomization (MR) is commonly used to assess causality in observational epidemiology and is frequently claimed to distinguish causal from non-causal associations. However, for complex phenotypes such as immune-mediated diseases, MR signals may arise from shared genetic architecture rather than direct exposure effects [6]. Even when MR confirms a (traditional) observational association, additional methods are needed to map the shared biology underlying to inform disease mechanisms and therapeutic development.

In this study, we used an integrated framework to interrogate the smoking–SjD paradox. Using MR, latent causal modelling, genome-wide pleiotropy analysis and colocalization, we assessed whether the observed association was more consistent with directional causality or with shared genetic architecture. We identified shared genetic loci influencing both traits and highlighted candidate genomic regions potentially relevant to immune regulation, epithelial biology and neuroimmune signalling. We then explored whether circulating proteins might help prioritize shared pathways linking smoking initiation liability and SjD risk. Together, this approach illustrates how paradoxical epidemiological associations can be leveraged for mechanistic hypothesis generation even when the observed association is more consistent with shared architecture than with directional causality.

## Methods

We performed an integrated cross-trait genetic analysis to investigate the relationship between smoking initiation liability (defined as ever becoming a regular smoker) and SjD using GWAS data. The analytical framework combined conventional MR, genome-wide causal modelling (CAUSE, latent causal variable analysis and linkage disequilibrium (LD) score regression), cross-trait pleiotropy mapping, locus-level colocalization and proteome-informed analyses to distinguish directional causality from shared genetic architecture and to identify candidate biological pathways underlying the smoking–SjD association. All GWAS datasets used in this study were obtained from previously published studies that had received appropriate institutional ethics approval and participant consent. No new individual-level data were collected for this analysis. MR reporting aligned with STROBE-MR recommendations [7] (checklist provided in Supplementary Tables).

### Smoking initiation (exposure)

Genetic associations for smoking were obtained from a large GWAS meta-analysis of 60 cohort studies and up to 3 383 199 individuals, using European-ancestry-specific summary statistics (*n* = 2 669 029) [8]. Smoking initiation was analysed as a binary phenotype indicating whether an individual had ever been a regular smoker, based on self-reported smoking behaviour. Across cohorts, this was ascertained using questionnaire-based measures such as having smoked over 100 cigarettes in a lifetime, having smoked daily for at least 1 month or having ever smoked regularly, with responses harmonized to a common ever or never definition. Throughout, ‘smoking initiation’ refers to the GWAS-defined phenotype of ever becoming a regular smoker, rather than brief experimentation with cigarettes or persistence/cessation among established smokers.

### SjD (outcome)

Genetic associations for SjD were obtained from a GWAS of European ancestry comprising 3232 cases and 17 481 population controls [9]. SjD cases were clinically defined across contributing cohorts using American-European Consensus Group (AECG) criteria.

### MR analyses

Single-nucleotide polymorphisms (SNPs) associated with smoking initiation at genome-wide significance (*P* < 5 × 10^−8^) were selected as instrumental variables. To ensure independence of instruments, variants were LD clumped at *r*^2^ < 0.001 within a 10 000 kb window, based on the European-ancestry reference panel from the 1000 Genomes Project [10].

A two-sample MR framework was used to estimate the effect of genetic liability to smoking initiation on SjD risk. The primary estimate was derived using inverse-variance weighted (IVW) MR under a multiplicative random-effects model, combining SNP-specific Wald ratios as a weighted average. Instrument strength was approximated using *F*-statistics calculated for each SNP as (*β*/SE)^2^, with *F* > 10 indicating sufficiently strong instruments. To reduce confounding from population stratification, analyses were restricted to European-ancestry GWAS for both exposure and outcome.

To evaluate the possibility of reverse causation as an explanation for any association observed in the primary MR analysis, we performed reverse-direction MR using SjD-associated variants (*P* < 5 × 10^−8^, *r*^2^ < 0.001) as instruments and smoking initiation as the outcome.

To assess robustness to directional horizontal pleiotropy (i.e. average bias arising from variant effects on the outcome through pathways unrelated to smoking initiation), we performed complementary MR estimators with differing assumptions. MR-Egger allows for a non-zero intercept to capture average directional pleiotropy. Weighted median provides a consistent causal estimate provided that at least 50% of the total weight comes from valid instruments. Weighted mode assumes that the largest cluster of instruments represents valid variants and yields a consistent estimate under this assumption. MR-PRESSO was additionally applied to identify and exclude outlier variants contributing to heterogeneity, providing a corrected causal estimate after outlier removal. A global test assessed the presence of significant pleiotropy across instruments, and a distortion test evaluated whether outlier removal meaningfully altered the causal estimate. These approaches provide statistical reassurance against some forms of pleiotropy but do not fully resolve bias arising from genome-wide correlated pleiotropy (shared genetic pathways affecting both traits), which we addressed using genome-wide modelling and locus-level analyses described below.

### Distinguishing causality from shared genetic architecture (CAUSE, LCV and LDSC)

Conventional MR can produce apparent exposure–outcome associations when genetic effects on the exposure and outcome are correlated across the genome due to shared biological pathways (correlated pleiotropy), rather than reflecting an exposure-mediated causal effect. We therefore applied two complementary, methodologically distinct genome-wide approaches to distinguish directional causality from shared genetic architecture.

CAUSE (Causal Analysis Using Summary Effect estimates) compares two genome-wide explanations for the observed genetic overlap. In the sharing model, there is no causal effect of the exposure on the outcome; instead, the association is explained by correlated horizontal pleiotropy arising from a shared heritable factor, together with uncorrelated horizontal pleiotropy. The causal model includes the same pleiotropic components but additionally allows an exposure-mediated causal effect on the outcome. The method evaluates which model better explains the pattern of genetic associations across the genome, thereby separating causal effects from correlated and uncorrelated pleiotropy. Independent variants were selected by pruning for LD at *r*^2^ < 0.01 within a 10 000 kb window [10], retaining SNPs associated with the exposure at *P* < 1 × 10^−3^ as recommended to maximize genome-wide information while limiting redundancy due to LD.

Latent causal variable (LCV) analysis provides a complementary genome-wide assessment of directionality. LCV models the shared genetic component of two traits using a latent variable and estimates the genetic causality proportion (GCP), which quantifies the extent to which the genetic component of one trait appears to be directionally causal for the other vs reflecting non-directional sharing. A GCP near +1 or −1 suggests a predominantly directional genetic causal effect (trait 1 → trait 2 or *vice versa*), and GCP near 0 suggests genetic correlation driven largely by shared architecture rather than directional causality. LCV therefore serves as an orthogonal check on whether genome-wide sharing is better explained by directionality or pleiotropy-driven overlap, complementing the model-comparison approach in CAUSE.

LD score regression (LDSC) was used to estimate genome-wide genetic correlation between smoking initiation liability and SjD. This provided a global measure of genetic overlap, complementing locus-level pleiotropy and colocalization analyses that can detect focal shared effects even in the absence of genome-wide correlation.

### Genome-wide pleiotropy mapping (PLACO)

Once shared architecture is plausible, the question remains which loci contribute to overlap between smoking initiation liability and SjD. PLACO (pleiotropic analysis under a composite null hypothesis) is designed to identify variants jointly associated with both traits, rather than forcing an exposure → outcome interpretation. PLACO tests a composite null where a variant affects neither trait, or only one trait, against the alternative that it influences both traits. In practice, it uses the product of *Z*-scores at each SNP from the two GWAS and evaluates significance under the composite null distribution. This approach is advantageous for detecting pleiotropy, particularly when genetic effects may be modest in one trait but consistently present across both. We excluded variants with MAF < 0.01 and extreme *Z*-scores (*Z*^2^ > 80) to reduce instability from rare alleles and very large effects. Genome-wide significance for pleiotropy was defined as *P* < 5 × 10^−8^.

### Locus definition and functional annotation (FUMA)

Variants demonstrating genome-wide evidence of pleiotropy were taken forward for locus definition and annotation using FUMA. Pleiotropic variants were grouped into genomic loci by clumping within a ±250 kb window. LD threshold of *r*^2^ < 0.6 was used to define independent significant variants within each locus, and *r*^2^ < 0.1 to identify lead SNPs (1000 Genomes Phase 3 reference panel). Lead SNPs were mapped to nearby protein-coding genes using positional mapping within 10 kb.

### Colocalization analysis

Bayesian colocalization was performed at pleiotropic loci to evaluate whether smoking initiation and SjD associations were consistent with a shared causal variant (hypothesis H4) or with distinct causal variants (hypothesis H3). For each locus, variants within ±200 kb of the lead SNP were extracted. Loci within the extended major histocompatibility complex (MHC) region (chr6: 25–34 Mb) were excluded due to extensive LD. Colocalization was conducted with default prior probabilities (*p*1 = 1 × 10^−4^ for association with smoking initiation, *p*2 = 1 × 10^−4^ for association with SjD and *p*12 = 1 × 10^−5^ for a shared causal variant). Evidence supporting colocalization was defined using the conditional posterior probability PPH4/(PPH3 + PPH4), with values >0.7 indicating support for a shared causal variant.

### Proteome-informed target nomination

To assess whether circulating proteins might mediate a potential causal pathway linking genetic liability to smoking initiation and SjD, we integrated genetic evidence on circulating proteins measured in the UK Biobank Pharma Proteomics Project (UKB-PPP), which quantified ∼3000 plasma proteins using the Olink Explore platform in ∼54 000 participants of predominantly European ancestry.

First, we estimated the association between genetic liability to smoking initiation and circulating-protein levels using two-sample MR, applying the same analytical framework as for smoking → SjD but treating each protein as the outcome. Multiple testing across proteins was controlled using false discovery rate (FDR) correction. Second, for proteins passing FDR, we tested whether genetically predicted protein levels were associated with SjD risk using cis-only instruments. Cis-pQTLs were defined as variants within ±200 kb of the encoding gene associated with protein abundance at *P* < 5 × 10^−6^, LD-clumped at *r*^2^ < 0.1 with variants in the extended MHC region excluded. For proteins instrumented by a single cis-pQTL, Wald ratios were used; for proteins with multiple cis instruments, fixed-effect IVW was applied. Protein → SjD results were FDR-corrected across tested proteins. Proteins passing FDR at stage 2 were further evaluated using Bayesian colocalization (as described above) to assess whether the protein and SjD association signals were consistent with a shared causal variant.

## Results

After genome-wide significance filtering and LD clumping, 93 independent SNPs were retained as instruments for smoking initiation. Instrument strength was high, with a median *F*-statistic of 35.45 and a minimum *F*-statistic of 29.8. The harmonized dataset included 90 SNPs after exclusion of palindromic, ambiguous and missing variants.

In the primary IVW MR analysis, genetic liability to smoking initiation was associated with 47% lower odds of SjD (OR 0.53, 95% CI 0.34, 0.84, *P* = 0.0063), consistent in direction with the reported epidemiological observation. The association was supported by pleiotropy-robust estimators: weighted median (OR 0.66, 95% CI 0.38, 1.16, *P* = 0.15), weighted mode (OR 0.60, 95% CI 0.13, 2.70, *P* = 0.51) and MR-Egger (OR 0.59, 95% CI 0.09, 3.76, *P* = 0.58) (Table 1 and Supplementary Fig. S1). There was no evidence of directional pleiotropy based on the MR-Egger intercept (−0.0013, *P* = 0.91). MR-PRESSO identified evidence of influential outlier variants, but the outlier-corrected estimate was consistent with the primary IVW result.

**Table 1:** Association between Sjögren’s disease and smoking initiation estimated using Mendelian randomization.

Outcome	Exposure	Method	No. SNPs	OR	Lower 95% CI	Upper 95% CI	*P*-value
Sjögren’s disease	Smoking initiation	Inverse variance weighted	218	0.53	0.34	0.84	0.0063
Sjögren’s disease	Smoking initiation	MR Egger	218	0.59	0.09	3.76	0.58
Sjögren’s disease	Smoking initiation	Weighted median	218	0.66	0.38	1.16	0.15
Sjögren’s disease	Smoking initiation	Weighted mode	218	0.60	0.13	2.70	0.51

Estimates represent odds ratios (ORs) for Sjögren’s disease per log-odds increase in genetic liability to smoking initiation.

SNPs, single-nucleotide polymorphisms.

Reverse-MR provided no evidence that genetic liability to SjD was associated with smoking initiation, with null estimates across IVW and sensitivity analyses (Supplementary Table S1).

### CAUSE

To contextualize MR findings within genome-wide genetic sharing, we applied CAUSE to compare a directional causal model with a sharing model for the observed genetic association. Model comparison provided no evidence that the causal model fit the data better than the sharing model (ΔELPD = −0.12, *P* = 0.45) (Supplementary Tables S2 and S3 and Fig. S2).

### LCV

Building on the CAUSE analysis, LCV modelling was used to assess whether shared genetic architecture exhibits a predominant directional effect. LCV provided no evidence of a directional genetic causal effect (GCP = −0.04, *P* = 0.96), indicating that the observed association was more consistent with non-directional shared genetic influences than with exposure-mediated causality.

### LDSC

LDSC showed no evidence of genome-wide correlation between smoking initiation liability and SjD (rG = −0.022, SE = 0.049, *P* = 0.660). This does not exclude locus-specific shared genetic effects, which were evaluated using PLACO and colocalization.

### PLACO

PLACO identified 1536 variants jointly associated with smoking initiation and SjD at genome-wide significance (*P* < 5 × 10^−8^). These variants mapped to 11 pleiotropic loci following LD-based clumping. Across loci, 16 independent lead SNPs were identified (Table 2). After adding GWAS beta coefficients and standard errors for both traits to Table 2, we observed that many pleiotropic lead variants had opposing directions of effect for smoking initiation and SjD. This pattern is directionally consistent with the inverse association observed in conventional MR, although individual SNP directions should be interpreted cautiously, particularly for loci without strong colocalization support.

**Table 2:** Lead SNPs at pleiotropic loci jointly associated with smoking initiation and Sjögren’s disease.

Locus	Lead SNP	Chr:Pos (hg19)	*P* (PLACO)	***β*** (smoking)	SE (smoking)	***β*** **(SjD)**	SE (SjD)	Nearest gene (summary)
1	rs77230051	2:145355508	3.56 × 10^−8^	0.015	0.002	−0.136	0.045	Intergenic
2	rs10187072	2:146101224	7.54 × 10^−9^	−0.016	0.002	−0.089	0.029	Intergenic
3	rs716936	3:117635198	7.93 × 10^−9^	0.017	0.002	−0.111	0.039	Near LSAMP
4	rs1549212	5:166996722	4.47 × 10^−8^	−0.016	0.002	−0.085	0.030	Within TENM2
5	rs927985	6:25412811	2.78 × 10^−8^	0.008	0.002	0.237	0.039	MHC region
6	rs1053860	6:26325228	1.62 × 10^−12^	−0.009	0.002	0.242	0.029	MHC region
7	rs1796518	6:26388672	3.48 × 10^−10^	−0.007	0.002	0.252	0.030	MHC region
8	rs4712987	6:26399615	1.29 × 10^−9^	−0.007	0.002	0.243	0.029	MHC region
9	rs13217239	6:27254967	3.12 × 10^−12^	−0.009	0.002	0.237	0.030	MHC region
10	rs2235233	6:27279852	3.24 × 10^−8^	0.007	0.002	−0.245	0.036	MHC region
11	rs3130814	6:29196181	1.20 × 10^−12^	0.008	0.002	0.260	0.030	MHC region
12	rs9277965	6:33256397	2.60 × 10^−9^	0.011	0.002	−0.211	0.043	MHC region
13	rs7844965	8:27442064	1.79 × 10^−8^	−0.017	0.002	0.092	0.034	Intergenic
14	rs61991652	14:77518204	3.41 × 10^−8^	0.011	0.002	−0.141	0.034	Near IRF2BPL
15	rs112538459	17:43527323	1.61 × 10^−9^	−0.010	0.002	−0.226	0.042	Intergenic
16	rs55938136	17:43798360	3.32 × 10^−8^	−0.008	0.002	−0.230	0.039	Intergenic

*P*-values correspond to the PLACO test for joint association. Genomic positions are reported in hg19 coordinates. Nearest genes are provided for annotation purposes only.

*β*, beta coefficient; SE, standard error; SNP, single-nucleotide polymorphism; PLACO, pleiotropic analysis under composite null hypothesis; hg19, human genome reference assembly GRCh37; MHC, major histocompatibility complex.

### FUMA

Using FUMA positional mapping, lead SNPs were annotated to nearby protein-coding genes within 10 kb, prioritizing candidate genes including *LSAMP*, *TENM2* and *IRF2BPL* (Table 2).

### Colocalization

After excluding loci in the extended MHC region, seven lead SNP regions were assessed for colocalization; five loci showed evidence consistent with a shared causal variant (conditional posterior probability >0.7, Supplementary Table S4). For the five colocalizing loci, colocalization support ranged from 0.735 to 0.983, with lead regions at 3q13.32, 2q22.3, 8p21.2, 14q24.3 and 5q34 (Supplementary Fig. S3). At the remaining loci, posterior support was inconclusive, suggesting either coincident but distinct signals or limited resolution due to LD and sample size.

### Exploratory proteome-informed mediation

Genetic liability to smoking initiation was associated with 862 circulating proteins at FDR < 0.05 in the proteome-wide MR analysis. When these proteins were evaluated for association with SjD using cis-instrument MR, none remained significant after correction for multiple testing (FDR < 0.05) providing no robust evidence that circulating proteins mediate the relationship between smoking initiation liability and SjD risk. However, 43 proteins showed nominal evidence of association with SjD (*P* < 0.05). Among these, 16 also showed evidence supportive of colocalization (conditional posterior probability > 0.8). These proteins are presented in Supplementary Table S5 as exploratory candidates for follow-up.

## Discussion

In this study, genetic liability to smoking initiation was inversely associated with SjD in conventional MR analyses, mirroring the existing body of observational evidence. However, genome-wide causal modelling did not support a directional exposure-mediated effect and instead suggested that the association is more consistent with shared genetic architecture. By combining genome-wide pleiotropy mapping with colocalization analyses, we identified several genomic regions jointly influencing smoking initiation liability and SjD, thereby generating mechanistic hypotheses for future study.

The inverse association between smoking and SjD has often been attributed to reverse causation or bias. Our findings support an additional explanation: smoking initiation liability and SjD may share genetic determinants, producing a reproducible association at the population level without requiring a protective exposure-mediated effect. Because the exposure reflects genetic liability to smoking initiation rather than smoking exposure itself, the results primarily inform shared genetic architecture between smoking behaviour and SjD susceptibility. Prior studies have reported differences in glandular inflammation, minor salivary gland biopsy positivity and serological features between smokers and non-smokers with SjD [11, 12]. Such mechanisms could contribute to cross-sectional differences within SjD cohorts through disease-course modification, measurement effects or selection bias, even if smoking initiation liability does not causally alter disease incidence. Collider bias may also contribute associations observed within sicca or SjD cohorts, particularly when analyses condition on factors influenced by both smoking-related traits and SjD-related biology, such as sicca symptoms, biopsy referral, serology or diagnosis. This may partly explain why inverse smoking-SjD associations appear stronger in studies using sicca controls than population controls. Because our genetic analyses used population controls, they do not directly address sicca-control contrasts, but these considerations reinforce caution in interpreting the observational association as a protective effect of smoking.

Although LDSC did not support substantial genome-wide genetic correlation, this does not preclude focal shared genetic effects at individual loci. Colocalization analyses provided locus-level resolution for such focal sharing, identifying five regions in which smoking initiation liability and SjD showed evidence consistent with a shared causal variant. These loci suggest candidate pathways involving neuroimmune signalling, epithelial biology and immune regulation. The 3q13.32 locus lies near *LSAMP*, making this gene a plausible candidate for future study [13]. The 5q34 locus maps within *TENM2*, a biologically plausible nearby candidate with functions in cell adhesion and tissue organization [14]. The 14q24.3 locus lies proximal to *IRF2BPL*, which is of interest given links to IRF-related regulation and the established importance of interferon signalling in SjD [15, 16]. The remaining colocalized regions at 2q22.3 and 8p21.2 are intergenic and may reflect regulatory mechanisms, although causal genes cannot be assigned with confidence from the present data. The predominance of discordant SNP-level effects among pleiotropic lead variants provides a locus-level correlate of the inverse MR association. However, because not all pleiotropic lead variants colocalized, we interpreted SNP directionality descriptively and prioritized biological interpretation of loci with evidence supporting a shared causal variant. Overall, these findings point to candidate upstream processes that merit follow-up in tissue- and cell-type-specific studies.

Exploratory proteome-informed analyses provided a complementary view of the shared architecture. We did not identify any circulating proteins that remained associated with SjD after correction for multiple testing in the protein → SjD analysis, and therefore found no robust evidence that circulating proteins mediate the relationship between smoking initiation liability and SjD risk. Nevertheless, 16 proteins showed nominal association with SjD together with colocalization support, representing hypothesis-generating signals for further investigation. Several of these proteins have biological relevance to SjD pathogenesis, including TNFSF13B (BAFF), a key regulator of B cell survival [17]; TNFRSF1B, involved in TNF signalling [18]; CD8A, reflecting cytotoxic T cell activity [19]; TNFRSF11A, which participates in NF-κB signalling [20] and FGR, an innate immune kinase implicated in myeloid cell activation [21]. These pathways are broadly consistent with established mechanisms of SjD, including dysregulated B cell responses and immune-mediated tissue inflammation. The direction of effect across exploratory protein candidates was heterogeneous: some proteins showed concordant associations with smoking liability and SjD, whereas others showed opposing directions. This pattern argues against a single circulating-protein mediator and is more consistent with multiple shared biological processes, some of which may act in opposing directions. These findings remain hypothesis-generating, as no protein → SjD association survived multiple-testing correction.

This study has several strengths. We applied multiple complementary causal inference methods, and integrated genome-wide modelling with locus-level colocalization to move beyond global genetic correlation. Limitations should also be noted. First, smoking initiation captures genetic liability to ever-smoking behaviour rather than cumulative exposure, intensity or cessation, and therefore may not capture the full biological consequences of sustained smoking exposure. Second, although CAUSE and LCV reduce bias from correlated pleiotropy, residual horizontal pleiotropy cannot be excluded. Third, analyses were restricted to European-ancestry datasets, which limits generalizability. Finally, although the SjD GWAS is among the largest currently available, its modest sample size may have limited power to detect small effects and to resolve causal variants precisely.

In summary, the long-standing inverse association between smoking and SjD was not supported here as a protective exposure-mediated effect of smoking initiation liability. Instead, the findings were more consistent with shared genetic architecture, with several loci highlighting candidate pathways for functional follow-up. More broadly, this study illustrates how paradoxical epidemiological associations can be used to generate mechanistic hypotheses when analysed using approaches that distinguish directional causality from shared genetic overlap. Future prospective studies with repeated smoking measures, sicca symptom assessment, objective glandular and serological phenotyping, and incident SjD outcomes would be valuable to disentangle exposure-mediated effects, disease-course modification and selection mechanisms.

## Supplementary Material

keag385_Supplementary_Data

## Data Availability

The GWAS summary statistics used in this study are publicly available. Smoking initiation summary statistics are available from the GWAS & Sequencing Consortium of Alcohol and Nicotine use (GSCAN). Sjögren’s disease GWAS summary statistics are available from the original publication [9].
