# Supplementary material for: From paradox to biology: shared genetic architecture underlies the inverse association between smoking and Sjögren’s disease
Source: Rheumatology (Oxford). 2026 Jul 24;65(8):keag385. doi: 10.1093/rheumatology/keag385 (PMC13433242; doi:10.1093/rheumatology/keag385)
Supplement: Supplementary file 1 [file keag385_supplementary_data.zip › rhe-26-0960-File002.docx]

**Supplementary tables for:**

From paradox to biology: shared genetic architecture underlies the inverse association between smoking and Sjögren’s disease

Stefanie van der Merwe^1^, Benjamin Zuckerman^1,2^, Weijie Liu^1^, Wenjie Cheng^1,3^, Sizheng Steven Zhao^1,4^

1. Centre for Musculoskeletal Research, Division of Musculoskeletal and Dermatological Science, School of Biological Sciences, Faculty of Biological Medicine and Health, The University of Manchester, Manchester Academic Health Science Centre, Manchester, UK

2. Centre for Rheumatic Diseases, Department of Inflammation Biology, King's College London, London, UK

3. Dermatology Center, Xinhua Hospital, Shanghai Jiaotong University School of Medicine, Shanghai, China

4. NIHR Manchester Biomedical Research Centre, Manchester University NHS Foundation Trust, Manchester, UK

Correspondence to: Sizheng S Zhao. Centre for Musculoskeletal Research, Division of Musculoskeletal and Dermatological Science, School of Biological Sciences, Faculty of Biological Medicine and Health, The University of Manchester, Manchester Academic Health Science Centre, Oxford Road, Manchester, M13 9LJ, UK. Email: [Sizheng.zhao@manchester.ac.uk](mailto:Sizheng.zhao@manchester.ac.uk). ORCiD iD: 0000-0002-3558-7353.

Contents

[Supplementary Table S1. Association between smoking initiation and Sjögren’s Disease estimated using reverse-direction Mendelian Randomisation (MR).	3](#_Toc231036564)

[Supplementary Table S2. Causal Analysis Using Summary Effect estimates (CAUSE) model comparison.	4](#_Toc231036565)

[Supplementary Table S3. Causal Analysis Using Summary Effect estimates (CAUSE) parameter estimates.	5](#_Toc231036566)

[Supplementary Table S4. Colocalisation analysis of pleiotropic loci for smoking initiation and Sjögren’s disease.	6](#_Toc231036567)

[Supplementary Table S5. Candidate proteins showing nominal association with Sjögren’s disease and supporting colocalisation.	7](#_Toc231036568)

[Supplementary Table S6. STROBE-MR Checklist of Recommended Items to Address in Reports of Mendelian Randomization (MR) Studies	8](#_Toc231036569)

| **Outcome** | **Exposure** | **Method** | **No. SNPs** | **OR** | **Lower 95% CI** | **Upper 95% CI** | **p-value** |
| --- | --- | --- | --- | --- | --- | --- | --- |
| Smoking initiation | Sjögren’s Disease | Inverse Variance Weighted | 13 | 1.00 | 0.99 | 1.01 | 0.90 |
| Smoking initiation | Sjögren’s Disease | MR Egger | 13 | 0.99 | 0.98 | 1.00 | 0.32 |
| Smoking initiation | Sjögren’s Disease | Weighted median | 13 | 1.00 | 0.99 | 1.00 | 0.09 |
| Smoking initiation | Sjögren’s Disease | Weighted mode | 13 | 1.00 | 0.99 | 1.00 | 0.10 |

**Supplementary Table S1. Association between smoking initiation and Sjögren’s Disease estimated using reverse-direction Mendelian Randomisation (MR).** Estimates represent odds ratios (ORs) for smoking initiation per log-odds increase in genetic liability to Sjögren’s disease. CI, confidence interval; SNPs, single nucleotide polymorphisms.

| **Model 1** | **Model 2** | **ΔELPD** | **SE (ΔELPD)** | **Z** | **p-value** |
| --- | --- | --- | --- | --- | --- |
| Null | Sharing | -0.39 | 1.10 | -0.35 | 0.36 |
| Null | Causal | -0.52 | 2.00 | -0.26 | 0.40 |
| Sharing | Causal | -0.12 | 0.91 | -0.14 | 0.45 |

**Supplementary Table S2. Causal Analysis Using Summary Effect estimates (CAUSE) model comparison.** ΔELPD represents the difference in expected log predictive density between models. SE denotes the standard error; Z and p-values assess statistical significance. The sharing model reflects correlated pleiotropy without causality, whereas the causal model permits an exposure-mediated effect.

| **Model** | **Gamma (causal effect)** | **Eta (shared effect)** | **Q (proportion sharing)** |
| --- | --- | --- | --- |
| Sharing | N/A | -1.85 (-5.38, 1.06) | 0.006 (0, 0.26) |
| Causal | -0.22 (-0.61, 0.19) | -0.11 (-4.23, 3.93) | 0.05 (0, 0.27) |

**Supplementary Table S3. Causal Analysis Using Summary Effect estimates (CAUSE) parameter estimates.** Gamma represents the causal effect estimate, eta represents the shared pleiotropic effect, and q represents the proportion of variants acting through the shared effect. Values in parentheses represent 95% credible intervals.

| **Genomic Locus** | **Lead SNP** | **H0** | **H1** | **H2** | **H3** | **H4** | **H4/(H4+H3)** |
| --- | --- | --- | --- | --- | --- | --- | --- |
| 3q13.32 | rs716936 | <0.001 | <0.001 | 0.553 | 0.021 | 0.426 | 0.954 |
| 2q22.3 | rs77230051 | <0.001 | <0.001 | 0.446 | 0.036 | 0.517 | 0.934 |
| 8p21.2 | rs7844965 | <0.001 | <0.001 | 0.712 | 0.076 | 0.211 | 0.735 |
| 14q24.3 | rs61991652 | 0.002 | 0.002 | 0.018 | 0.017 | 0.961 | 0.983 |
| 5q34 | rs1549212 | <0.001 | <0.001 | 0.808 | 0.049 | 0.143 | 0.744 |
| 17q21.31 | rs112538459 | <0.001 | 0.180 | 0.000 | 0.390 | 0.430 | 0.524 |
| 2q22.3 | rs10187072 | <0.001 | <0.001 | 0.886 | 0.080 | 0.033 | 0.294 |

Supplementary Table S4. Colocalisation analysis of pleiotropic loci for smoking initiation and Sjögren’s disease. H0-H4 represent posterior probabilities for the following hypotheses: H0, neither trait has a genetic association in the region; H1, only trait 1 has a genetic association; H2, only trait 2 has a genetic association; H3, both traits are associated but with different causal variants; H4, both traits are associated and share a single causal variant. H4/(H3+H4) represents the probability of colocalisation conditional on the presence of a causal variant for the outcome. SNP, single nucleotide polymorphism.

| **Protein** | **No. SNPs (smoking -> protein)** | **β (smoking -> protein)** | **P-value (smoking -> protein)** | **FDR (smoking -> protein)** | **No. SNPs (protein -> SjD)** | **β (protein -> SjD)** | **P-value (protein -> SjD)** | **FDR (protein -> SjD)** | **No. SNPs (colocalisation region)** | **PP.H1** | **PP.H2** | **PP.H3** | **PP.H4** |
| --- | --- | --- | --- | --- | --- | --- | --- | --- | --- | --- | --- | --- | --- |
| OMG | 485 | -0.245 | 1.52E-13 | 1.01E-11 | 2 | -0.781 | 0.016 | 0.664 | 204 | 0.823 | 7.58E-29 | 0.006 | 0.171 |
| LRRN1 | 485 | -0.214 | 2.6E-11 | 1.15E-09 | 39 | 0.124 | 0.001 | 0.476 | 776 | 0.263 | 0E+00 | 0.024 | 0.712 |
| F7 | 485 | 0.113 | 8.24E-04 | 0.005 | 16 | 0.141 | 0.008 | 0.55 | 396 | 0.693 | 0E+00 | 0.012 | 0.295 |
| FGR | 485 | 0.089 | 0.012 | 0.041 | 2 | 0.64 | 0.043 | 0.664 | 128 | 0.857 | 4.74E-28 | 0.007 | 0.136 |
| TNFRSF11A | 485 | 0.102 | 0.001 | 0.006 | 32 | -0.141 | 0.003 | 0.55 | 774 | 0.464 | 0E+00 | 0.032 | 0.504 |
| IGFBP4 | 485 | 0.24 | 4.49E-12 | 2.34E-10 | 1 | -1.649 | 0.022 | 0.664 | 224 | 0.813 | 1.99E-04 | 0.013 | 0.161 |
| NTRK3 | 485 | -0.321 | 1.46E-18 | 2.67E-16 | 20 | -0.232 | 0.034 | 0.664 | 647 | 0.62 | 1.62E-139 | 0.029 | 0.351 |
| TNFRSF1B | 485 | 0.147 | 5.41E-05 | 4.48E-04 | 4 | -0.375 | 0.034 | 0.664 | 323 | 0.828 | 4E-91 | 0.016 | 0.156 |
| ENPP7 | 485 | 0.155 | 2.03E-07 | 3.65E-06 | 11 | -0.067 | 0.032 | 0.664 | 164 | 0.916 | 0E+00 | 0.008 | 0.076 |
| TNFSF13B | 485 | 0.185 | 4.43E-07 | 7.12E-06 | 9 | 0.327 | 0.01 | 0.55 | 614 | 0.727 | 1.89E-93 | 0.026 | 0.247 |
| CGREF1 | 485 | 0.097 | 0.003 | 0.015 | 15 | 0.129 | 0.008 | 0.55 | 262 | 0.854 | 0E+00 | 0.015 | 0.131 |
| SMPD3 | 485 | -0.114 | 0.002 | 0.008 | 1 | 1.433 | 0.029 | 0.664 | 309 | 0.88 | 5.16E-05 | 0.014 | 0.103 |
| CD8A | 485 | 0.116 | 0.002 | 0.011 | 6 | 0.228 | 0.017 | 0.664 | 194 | 0.903 | 2.03E-286 | 0.014 | 0.083 |
| RAB44 | 485 | 0.088 | 0.011 | 0.04 | 11 | -0.308 | 0.038 | 0.664 | 630 | 0.827 | 1.66E-45 | 0.026 | 0.146 |
| KLK13 | 485 | 0.11 | 0.001 | 0.006 | 12 | 0.208 | 0.048 | 0.664 | 507 | 0.81 | 0E+00 | 0.03 | 0.16 |
| SUSD5 | 485 | -0.195 | 3.82E-10 | 1.36E-08 | 24 | -0.097 | 0.05 | 0.664 | 596 | 0.816 | 0E+00 | 0.036 | 0.148 |

**Supplementary Table S5. Candidate proteins showing nominal association with Sjögren’s disease and supporting colocalisation.** Beta (β) coefficients represent effect estimates from Mendelian randomisation analyses of smoking initiation on protein levels and protein levels on Sjögren’s disease (SjD). SNPs, single nucleotide polymorphisms; FDR, false discovery rate. PP.H1-H4 denote posterior probabilities from colocalisation analyses.

**Supplementary Table S6. STROBE-MR Checklist of Recommended Items to Address in Reports of Mendelian Randomization (MR) Studies**

| **Item No.** | **Section** | **Checklist item** | **Page No.** | **Relevant text from manuscript** |
| --- | --- | --- | --- | --- |
| 1 | **TITLE and ABSTRACT** | Indicate Mendelian randomization (MR) as the study’s design in the title and/or the abstract if that is a main purpose of the study | 2 | Mendelian randomization study in abstract |
|  | **INTRODUCTION** |  |  |  |
| 2 | **Background** | Explain the scientific background and rationale for the reported study. What is the exposure? Is a potential causal relationship between exposure and outcome plausible? Justify why MR is a helpful method to address the study question | 3 | Exposure: Genetic liability to smoking initiation;  Outcome: Sjögren’s disease (SjD) |
| 3 | **Objectives** | State specific objectives clearly, including pre-specified causal hypotheses (if any). State that MR is a method that, under specific assumptions, intends to estimate causal effects | 3 | Is genetically proxied smoking initiation causally associated with SjD |
|  | **METHODS** |  |  |  |
| 4 | **Study design and data sources** | Present key elements of the study design early in the article. Consider including a table listing sources of data for all phases of the study. For each data source contributing to the analysis, describe the following: |  |  |
|  | a) | Setting: Describe the study design and the underlying population, if possible. Describe the setting, locations, and relevant dates, including periods of recruitment, exposure, follow-up, and data collection, when available. | 4 | Detailed methods with references provided. |
|  | b) | Participants: Give the eligibility criteria, and the sources and methods of selection of participants. Report the sample size, and whether any power or sample size calculations were carried out prior to the main analysis | 4 | Detailed methods with references provided |
|  | c) | Describe measurement, quality control and selection of genetic variants | 4 | Detailed methods with references provided |
|  | d) | For each exposure, outcome, and other relevant variables, describe methods of assessment and diagnostic criteria for diseases | 4 | Detailed methods with references provided |
|  | e) | Provide details of ethics committee approval and participant informed consent, if relevant | 4 | Ethical approval had been obtained in all original studies |
| 5 | **Assumptions** | Explicitly state the three core IV assumptions for the main analysis (relevance, independence and exclusion restriction) as well assumptions for any additional or sensitivity analysis | 4-5 | Detailed discussion in methods |
| 6 | **Statistical methods: main analysis** | Describe statistical methods and statistics used |  |  |
|  | a) | Describe how quantitative variables were handled in the analyses (i.e., scale, units, model) | 4-5 | Presented as odds ratios for SjD per log-odds increase in liability to smoking initiation |
|  | b) | Describe how genetic variants were handled in the analyses and, if applicable, how their weights were selected | n/a |  |
|  | c) | Describe the MR estimator (e.g. two-stage least squares, Wald ratio) and related statistics. Detail the included covariates and, in case of two-sample MR, whether the same covariate set was used for adjustment in the two samples | 4 | Wald ratio |
|  | d) | Explain how missing data were addressed | n/a |  |
|  | e) | If applicable, indicate how multiple testing was addressed | 4-7 | FDR correction was applied to protein analyses; genome-wide significance set at P < 5 x 10^-8^ |
| 7 | **Assessment of assumptions** | Describe any methods or prior knowledge used to assess the assumptions or justify their validity | 4-5 | Assumptions discussed in methods |
| 8 | **Sensitivity analyses and additional analyses** | Describe any sensitivity analyses or additional analyses performed (e.g. comparison of effect estimates from different approaches, independent replication, bias analytic techniques, validation of instruments, simulations) | 4-7 | MR-Egger, weighted median, weighted mode for sensitivity analyses, CAUSE, LCV, PLACO, colocalisation for shared genetic architecture |
| 9 | **Software and pre-registration** |  |  |  |
|  | a) | Name statistical software and package(s), including version and settings used | n/a |  |
|  | b) | State whether the study protocol and details were pre-registered (as well as when and where) | n/a |  |
|  | **RESULTS** |  |  |  |
| 10 | **Descriptive data** |  |  |  |
|  | a) | Report the numbers of individuals at each stage of included studies and reasons for exclusion. Consider use of a flow diagram | n/a | Not applicable for summary statistics. |
|  | b) | Report summary statistics for phenotypic exposure(s), outcome(s), and other relevant variables (e.g. means, SDs, proportions) | n/a | Not applicable for summary statistics |
|  | c) | If the data sources include meta-analyses of previous studies, provide the assessments of heterogeneity across these studies | 4 | Not feasible to list all details within the word limit, but references are provided. |
|  | d) | For two-sample MR:  i.  Provide justification of the similarity of the genetic variant-exposure associations between the exposure and outcome samples  ii.  Provide information on the number of individuals who overlap between the exposure and outcome studies | 4 | (i) Exposure and outcome GWAS restricted to European ancestry populations  (ii) Negligible |
| 11 | **Main results** |  |  |  |
|  | a) | Report the associations between genetic variant and exposure, and between genetic variant and outcome, preferably on an interpretable scale | 16 | Details in Table 1 |
|  | b) | Report MR estimates of the relationship between exposure and outcome, and the measures of uncertainty from the MR analysis, on an interpretable scale, such as odds ratio or relative risk per SD difference | n/a |  |
|  | c) | If relevant, consider translating estimates of relative risk into absolute risk for a meaningful time period | n/a |  |
|  | d) | Consider plots to visualize results (e.g. forest plot, scatterplot of associations between genetic variants and outcome versus between genetic variants and exposure) | n/a |  |
| 12 | **Assessment of assumptions** |  |  |  |
|  | a) | Report the assessment of the validity of the assumptions | 4-7 | Assessed whether MR results reflect correlated pleiotropy rather than causality |
|  | b) | Report any additional statistics (e.g., assessments of heterogeneity across genetic variants, such as *I^2^*, Q statistic or E-value) | n/a |  |
| 13 | **Sensitivity analyses and additional analyses** |  |  |  |
|  | a) | Report any sensitivity analyses to assess the robustness of the main results to violations of the assumptions | 8 |  |
|  | b) | Report results from other sensitivity analyses or additional analyses | 8-9 |  |
|  | c) | Report any assessment of direction of causal relationship (e.g., bidirectional MR) | 8 |  |
|  | d) | When relevant, report and compare with estimates from non-MR analyses | 9-12 |  |
|  | e) | Consider additional plots to visualize results (e.g., leave-one-out analyses) | n/a | Not included |
|  | **DISCUSSION** |  |  |  |
| 14 | **Key results** | Summarize key results with reference to study objectives | 9-12 |  |
| 15 | **Limitations** | Discuss limitations of the study, taking into account the validity of the IV assumptions, other sources of potential bias, and imprecision. Discuss both direction and magnitude of any potential bias and any efforts to address them | 11 |  |
| 16 | **Interpretation** |  |  |  |
|  | a) | Meaning: Give a cautious overall interpretation of results in the context of their limitations and in comparison with other studies | 9-12 |  |
|  | b) | Mechanism: Discuss underlying biological mechanisms that could drive a potential causal relationship between the investigated exposure and the outcome, and whether the gene-environment equivalence assumption is reasonable. Use causal language carefully, clarifying that IV estimates may provide causal effects only under certain assumptions | 9-12 |  |
|  | c) | Clinical relevance: Discuss whether the results have clinical or public policy relevance, and to what extent they inform effect sizes of possible interventions | 9-12 |  |
| 17 | **Generalizability** | Discuss the generalizability of the study results (a) to other populations, (b) across other exposure periods/timings, and (c) across other levels of exposure | 11-12 |  |
|  | **OTHER INFORMATION** |  |  |  |
| 18 | **Funding** | Describe sources of funding and the role of funders in the present study and, if applicable, sources of funding for the databases and original study or studies on which the present study is based | 13 |  |
| 19 | **Data and data sharing** | Provide the data used to perform all analyses or report where and how the data can be accessed, and reference these sources in the article. Provide the statistical code needed to reproduce the results in the article, or report whether the code is publicly accessible and if so, where | 13 |  |
| 20 | **Conflicts of Interest** | All authors should declare all potential conflicts of interest | 13 |  |
