# Supplementary material for: From paradox to biology: shared genetic architecture underlies the inverse association between smoking and Sjögren’s disease
Source: Rheumatology (Oxford). 2026 Jul 24;65(8):keag385. doi: 10.1093/rheumatology/keag385 (PMC13433242; doi:10.1093/rheumatology/keag385)
Supplement: Supplementary file 1 [file keag385_supplementary_data.zip › rhe-26-0960-File003.pdf]

Supplementary figures for:

From paradox to biology: shared genetic architecture underlies the inverse association between smoking and Sjögren’s disease

Stefanie van der Merwe[1], Benjamin Zuckerman[1,2], Weijie Liu[1], Wenjie Cheng[1,3], Sizheng Steven Zhao[1,4]

1. Centre for Musculoskeletal Research, Division of Musculoskeletal and Dermatological Science, School of Biological Sciences, Faculty of Biological Medicine and Health, The University of Manchester, Manchester Academic Health Science Centre, Manchester, UK

2. Centre for Rheumatic Diseases, Department of Inflammation Biology, King's College London, London, UK

3. Dermatology Center, Xinhua Hospital, Shanghai Jiaotong University School of Medicine, Shanghai, China

4. NIHR Manchester Biomedical Research Centre, Manchester University NHS Foundation Trust, Manchester, UK

Correspondence to: Sizheng S Zhao. Centre for Musculoskeletal Research, Division of Musculoskeletal and Dermatological Science, School of Biological Sciences, Faculty of Biological Medicine and Health, The University of Manchester, Manchester Academic Health Science Centre, Oxford Road, Manchester, M13 9LJ, UK. Email: Sizheng.zhao@manchester.ac.uk. ORCiD iD: 0000-0002-3558-7353.

Contents

Supplementary Figure S1. Scatter plot of SNP effects on smoking initiation versus Sjögren's disease. 3

Supplementary Figure S2. Causal Analysis Using Summary Effect estimates (CAUSE) model comparison plots. 4

Supplementary Figure S3. Regional colocalisation of smoking initiation and Sjögren's disease association signals. 6

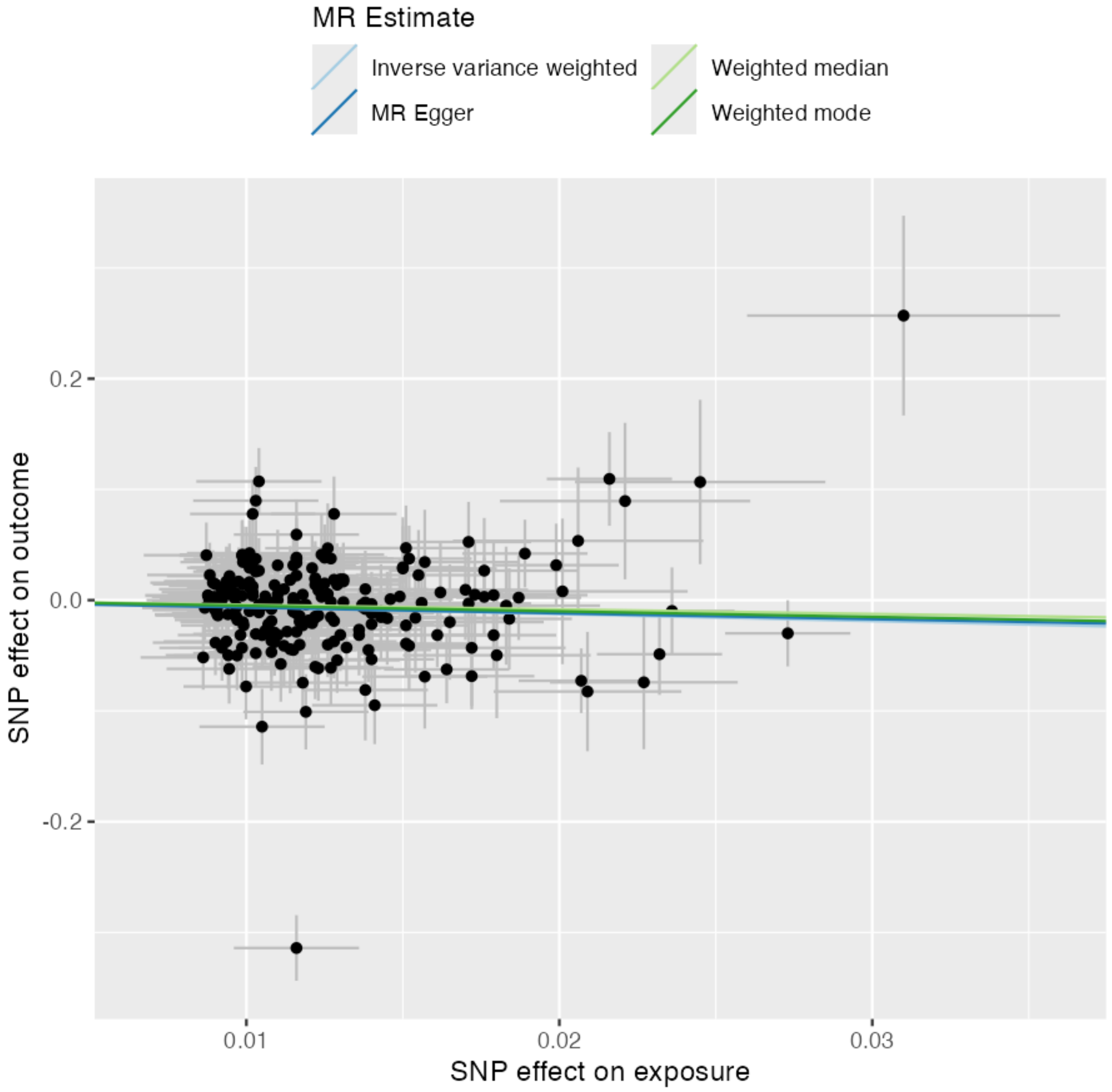

Supplementary Figure S1. Scatter plot of SNP effects on smoking initiation versus Sjögren's disease. Scatter plot of SNP effects on smoking initiation (exposure) versus Sjögren's disease (outcome). Each point represents a genetic instrument, with horizontal and vertical bars indicating standard errors. Lines correspond to causal effect estimates from the inverse-variance weighted, MR-Egger, weighted median, and weighted mode methods. SNP, single nucleotide polymorphism; MR, Mendelian randomisation.

Alt text: Scatter plot showing effect estimates for each genetic instrument on smoking initiation on the x-axis and Sjögren's disease on the y-axis, with four regression lines from inverse-variance weighted, Mendelian randomisation-Egger, weighted median, and weighted mode methods showing a slightly negative slope.

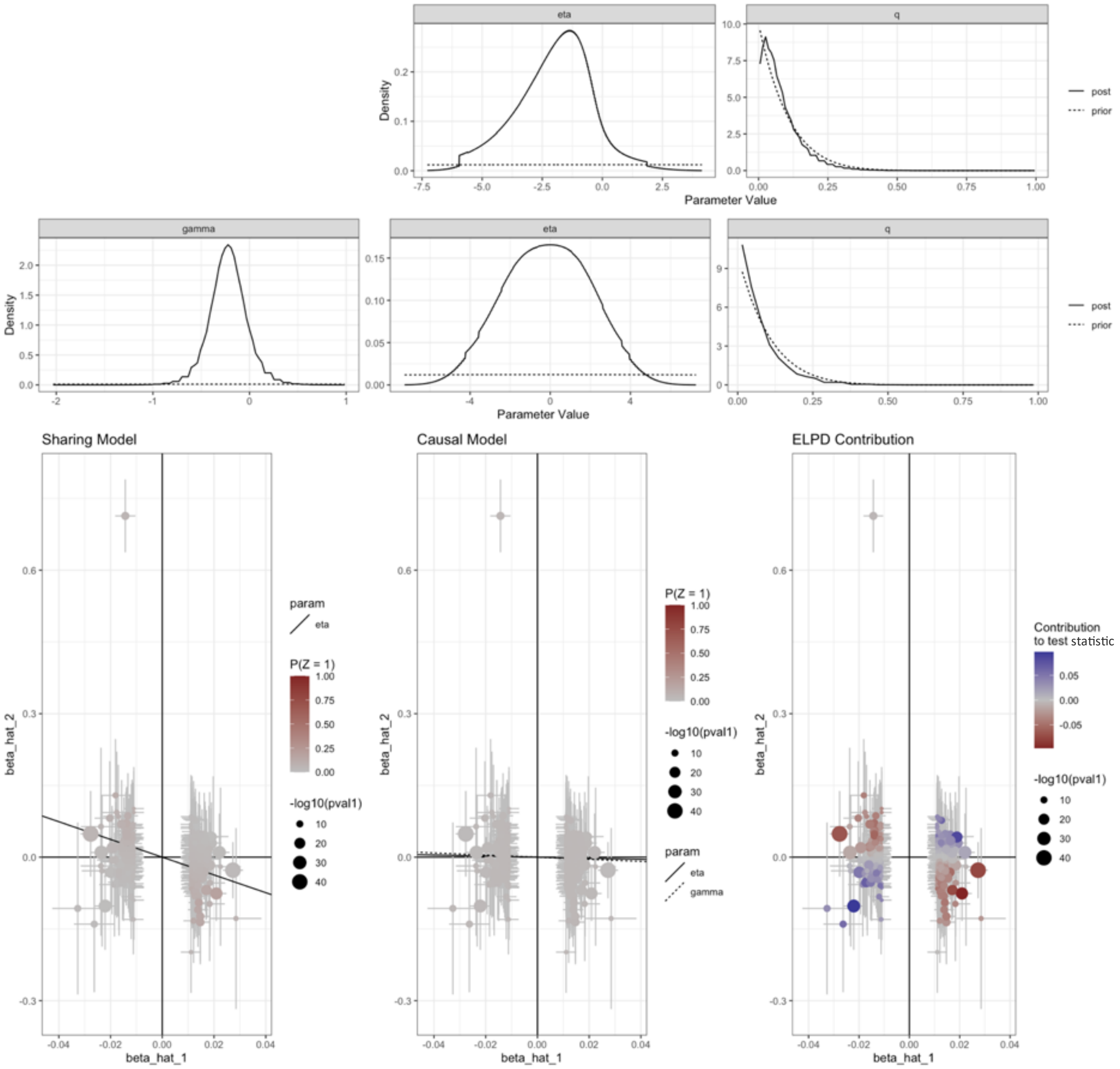

Supplementary Figure S2. Causal Analysis Using Summary Effect estimates (CAUSE) model comparison plots. Top: Posterior distributions of the CAUSE model parameters (gamma, eta, q) under the sharing and causal models, showing the posterior (solid line) and prior (dashed line). Bottom left: Variant effect estimates under the sharing model with posterior probability of acting through the shared effect (P(Z=1)). Bottom middle: Variant effect estimates under the causal model showing estimated causal (gamma) and shared (eta) effects. Bottom right: Variant-level contributions to the expected log predictive density (ELPD) difference between models.

Alt text: Multi-panel figure showing posterior distributions of Causal Analysis Using Summary Effect estimates model parameters under sharing and causal models in the top two rows, and variant-level effect estimates and expected log predictive density contributions in the bottom row.

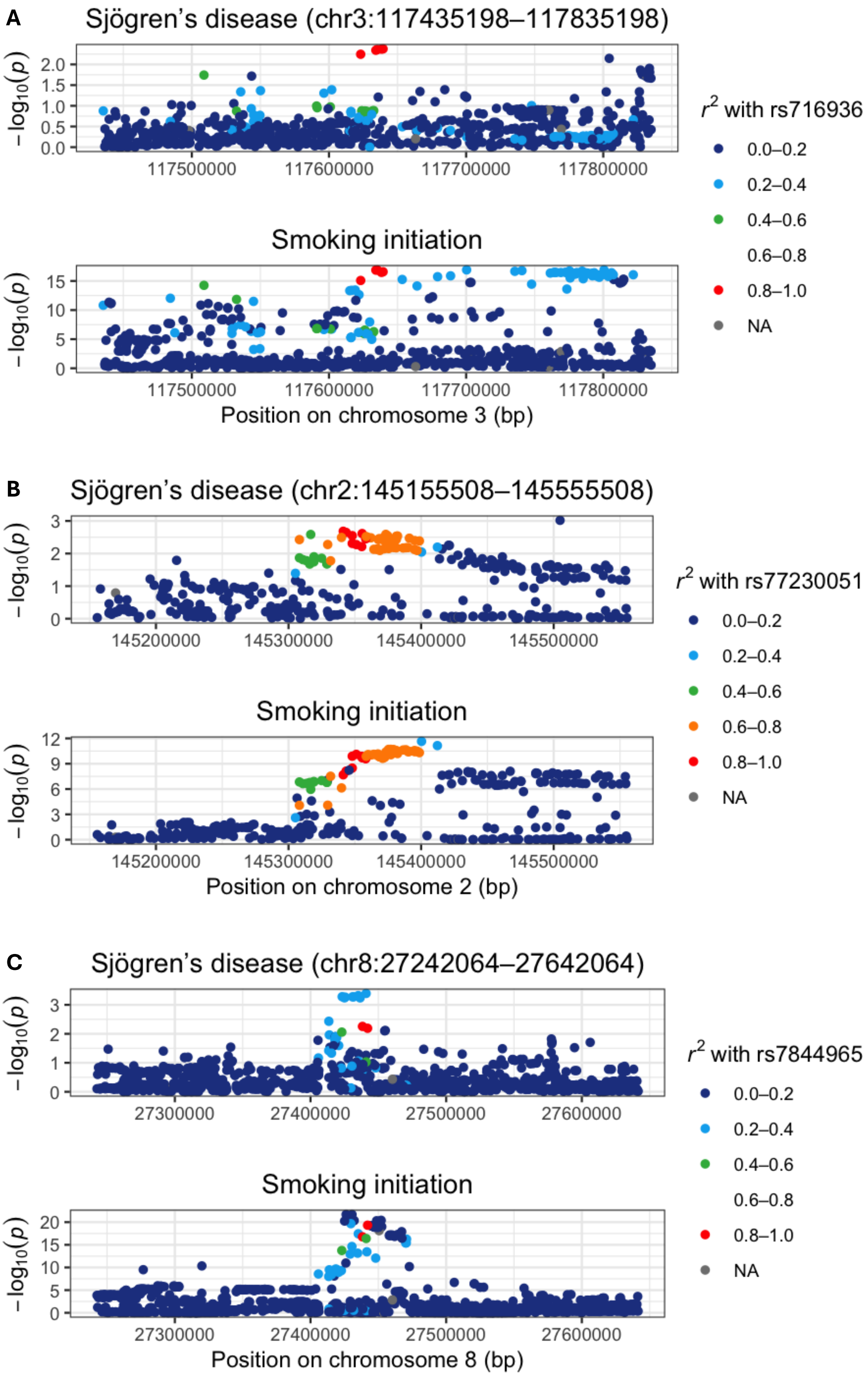

A
Sjögren's disease (chr3:117435198–117835198)
−log10(p)
2.0
1.5
1.0
0.5
0.0
117500000
117600000
117700000
117800000
Smoking initiation
15
10
5
0
Position on chromosome 3 (bp)
r2 with rs716936
0.0–0.2
0.2–0.4
0.4–0.6
0.6–0.8
0.8–1.0
NA
B
Sjögren's disease (chr2:145155508–145555508)
3
2
1
0
145200000
145300000
145400000
145500000
Smoking initiation
12
9
6
3
0
Position on chromosome 2 (bp)
r2 with rs77230051
C
Sjögren's disease (chr8:27242064–27642064)
27300000
27400000
27500000
27600000
Smoking initiation
20
15
10
5
0
Position on chromosome 8 (bp)
r2 with rs7844965

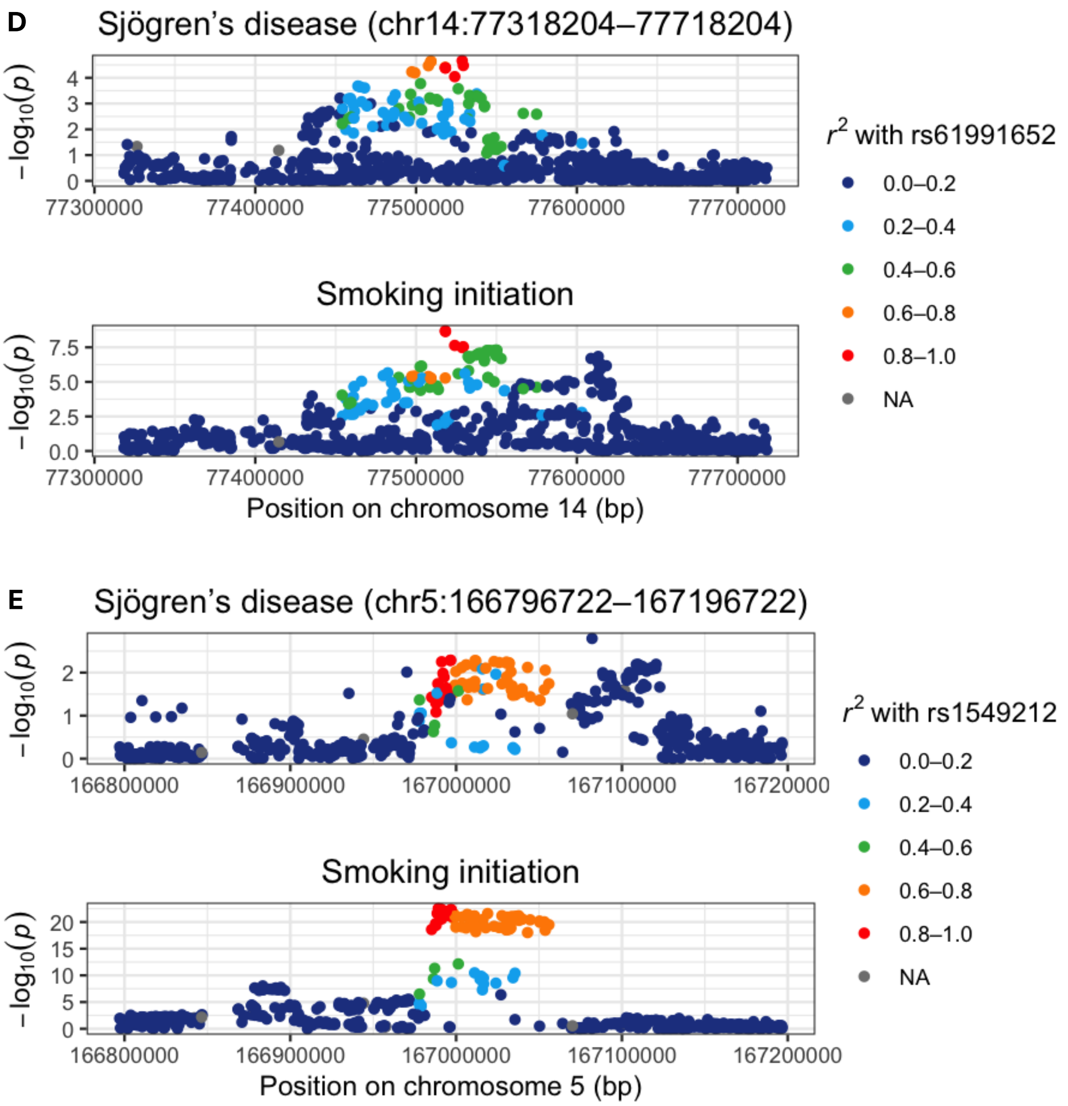

Supplementary Figure S3. Regional colocalisation of smoking initiation and Sjögren's disease association signals. Plots illustrate colocalised genetic signals at five loci: (A) 3q13.32, (B) 2q22.3, (C) 8p21.2, (D) 14q24.3, and (E) 5q34. For each locus, association results for Sjögren's disease (top panel) and smoking initiation (bottom panel) are shown as -log10(p) values plotted against genomic position (hg19). Points represent individual variants within each region and are coloured according to linkage disequilibrium (r^2) with the lead SNP, as indicated in the legend. Concordant association peaks and shared LD structure across traits are consistent with colocalisation at these loci. SNP, single nucleotide polymorphism; LD, linkage disequilibrium; hg19, human genome reference assembly GRCh37.

Alt text: Multi-panel figure with five regional association plots labelled A to E, each showing negative log10 p-values for Sjögren's disease and smoking initiation plotted against genomic position. Points are coloured by linkage disequilibrium with the lead variant, with concordant association peaks visible at each locus.
